# The First COVID-19 Stay-at-Home Restrictions: An Intergenerational Comparison of the Impacts

**DOI:** 10.1093/geroni/igaa057.3504

**Published:** 2020-12-16

**Authors:** Anne Glass, Lauretta Lawlor

**Affiliations:** University of North Carolina Wilmington, Wilmington, North Carolina, United States

## Abstract

“Social distancing” and stay-at-home orders were enacted in many states in response to the spread of COVID-19. We sought to understand intergenerational differences in the impact of the initial COVID-19 restrictions on interactions, loneliness, and stress. Data was collected via online survey from individuals ages 18 and above during the period April 7-May 8, 2020. The predominantly female, White, and well-educated sample (n = 962) included 245 younger adults (YAs) ages 18-34, 374 middle-aged adults (MAs) ages 35-64, and 343 older adults (OAs) ages 65+, with overall mean age 51.67 (SD=20.257; range 18-96). Before the restrictions, 41% of these OAs infrequently/never interacted face-to-face with children, increasing to 74% after restrictions. Three quarters (77%) of YAs reported seeing OAs less often, but 42% reported increasing their connections with OAs via technology. About a third of MAs (35%) and OAs (36%) were lonely, compared to 48% of YAs (p = .003), and a higher percentage of YAs (57%) reported being “more lonely” now, compared to MAs (36%) and OAs (41%). OAs reported the least stress; 42% reported low/very low levels of stress compared to YAs (9%) and MAs (20%). All generations most often identified “being able to go places” as the thing they missed most, but it increased significantly with age (p < .001), from YAs (32%), MAs (37%), to OAs (44%). More YAs (20%) than OAs (7%) reported missing “structure to their day.” Results of these intergenerational comparisons suggest the resilience of older adults is helping them during the current pandemic.

